# DeepNEU: cellular reprogramming comes of age – a machine learning platform with application to rare diseases research

**DOI:** 10.1186/s13023-018-0983-3

**Published:** 2019-01-10

**Authors:** Wayne R. Danter

**Affiliations:** 123Genetix, 147 Chesham Ave, London, ON N6G 3V2 Canada

**Keywords:** iPSCs, Cellular reprogramming, Machine learning, Neutrosophic and fuzzy cognitive maps, Recurrent neural network, RNN

## Abstract

**Background:**

Conversion of human somatic cells into induced pluripotent stem cells (iPSCs) is often an inefficient, time consuming and expensive process. Also, the tendency of iPSCs to revert to their original somatic cell type over time continues to be problematic. A computational model of iPSCs identifying genes/molecules necessary for iPSC generation and maintenance could represent a crucial step forward for improved stem cell research. The combination of substantial genetic relationship data, advanced computing hardware and powerful nonlinear modeling software could make the possibility of artificially-induced pluripotent stem cells (aiPSC) a reality. We have developed an unsupervised deep machine learning technology, called DeepNEU that is based on a fully-connected recurrent neural network architecture with one network processing layer for each input. DeepNEU was used to simulate aiPSC systems using a defined set of reprogramming transcription factors. Genes/proteins that were reported to be essential in human pluripotent stem cells (hPSC) were used for system modelling.

**Results:**

The Mean Squared Error (MSE) function was used to assess system learning. System convergence was defined at MSE < 0.001. The markers of human iPSC pluripotency (*N* = 15) were all upregulated in the aiPSC final model. These upregulated/expressed genes in the aiPSC system were entirely consistent with results obtained for iPSCs.

**Conclusion:**

This research introduces and validates the potential use of aiPSCs as computer models of human pluripotent stem cell systems. Disease-specific aiPSCs have the potential to improve disease modeling, prototyping of wet lab experiments, and prediction of genes relevant and necessary for aiPSC production and maintenance for both common and rare diseases in a cost-effective manner.

## Background

### Cellular reprogramming and modeling of human diseases

#### Advances in cellular reprogramming

The field of cellular reprogramming has evolved rapidly since mid-twentieth century. In the 1950s, the earliest attempts of cloning used a frog embryonic model [1]. Cloning was subsequently refined through somatic cell nuclear transplantation (SCNT) of the differentiated cells [2]. In the 1990s, advances in the field continued to emerge and, following substantial fine-tuning, led to successful cloning of the first mammal (Dolly the sheep) [3]. More recently, Yamanaka’s group showed that they could turn back the differentiation clock of somatic fibroblasts, first in mice [4], and then in humans [5, 6]. Their advance was achieved through the induced overexpression of just four key transcription factors (Oct4, Sox2, Klf4 and c-Myc) to generate embryonic stem-like cells, which were later referred to as induced pluripotent stem cells (iPSCs) [4–6]. In 2012, professor Yamanaka won the Nobel prize for his contribution to the field of cellular reprogramming and regenerative medicine.

#### Modeling human disease

Disease modeling is an essential tool to elucidate the molecular basis of numerous pathologies and enable development of novel targeted therapies. Several approaches are currently used to model human disease, including culture of primary patient-derived cells and over-expression of transfected genes correlated with disease in pre-identified cell culture lineage and/or animal models [7, 8]. However, there are limitations associated with each of these disease-modeling approaches. For example, the use of primary human cells is limited by (1) access to donors, especially in rare diseases (2) difficulty in gaining access to cells from certain organs (e.g. neuronal and cardiac cells) and (3) the short life span and/or ex vivo proliferative capacity of these cells. Additionally, transgene over-expression does not faithfully reflect physiological and pathological conditions. Finally, the differences between animal and human genomes, physiology, and patterns of gene expression make it challenging to translate findings obtained from animal modeling to clinical settings [8–10]. Thanks to the development of iPSCs, it is now possible to isolate somatic cells from patients and reprogram these cells into almost any specific cell lineage with the desired genetic background. The concept of “disease in a dish” using iPSCs has created new opportunities for experimentally-derived understanding of the underlying mechanisms of disease leading to new targeted therapeutic options. However, use of iPSC technologies has been successful in modelling some diseases and not in others.

### Deep-machine learning to enable efficient disease modeling

*iPSCs for modeling disease and current challenges* − Since the generation of iPSCs from human fibroblasts [6], the technology has advanced rapidly. iPSC-based disease models have been developed for numerous diseases affecting different human systems, including neurological, cardiovascular, hematological, metabolic, epigenetic, telomere and mitochondrial diseases and more [11–15]. Despite advances in iPSC technology, the production of these iPSCs continues to be limited by the lack of efficient induction protocols [16–18]. In fact, the average efficiency of human pluripotent stem cell (PSC) induction protocols ranges from 0.001–1.0% based on reprogramming method and cell lineage and is usually dependent on experimental conditions [16, 18]. Other ongoing issues include cost/resource requirements and tendency of iPSCs to return to the genetic makeup of the original somatic cell type over time [19–21]. Such limitations in the current cellular reprogramming methods underscore the need for improved stem cell generation strategies.

#### Deep-machine learning for efficient iPSC modeling

Elucidating the underlying mechanisms of cellular reprogramming is still at an early stage of understanding. Nonetheless, extensive and ongoing research has produced new methods for improving the efficiency of iPSC generation. For example, several studies have investigated the effect of small molecules on the efficiency of various PSC induction protocols. Others focus on evaluating the association between the level of expressed pluripotent transcription factors and the efficiency of inducting protocols for PSCs [18, 22–24]. However, there is increasing demand for fast, accurate, deep, and cost-effective analytical approaches to effectively enable iPSC-based model generation and subsequent modelling of human diseases, including rare ones where access to patient-derived primary somatic cells is very limited. In this study, we introduce a novel unsupervised deep-machine learning platform, called DeepNEU, to simulate iPSCs and enable efficient cellular reprogramming. We have validated the DeepNEU platform extensively, as presented in the current work. The platform has been employed and validated by developing computer simulations of three iPSCs models that were previously generated experimentally and published in the peer-reviewed literature [6, 25–27]. Here we have generated models of artificially-induced pluripotent stem cells (aiPSCs), artificially-induced neural stem cells (aiNSCs) and artificially-induced cardiomyocytes (aiCMCs). Additionally, the aiNSC model has been used to successfully simulate a rare neurological disorder, Rett syndrome, that is caused by methyl-CpG-binding protein 2 (MeCP2) deficiency in about 80% of cases [28].

## Results

### DeepNEU platform specification

The DeepNEU database (Version 3.2) contains 3589 gene/proteins (~ 10% of the human genome) and 27,566 nonzero relationships resulting in a large amount of information flowing into and out of each node in the network. On average, each node in the network has more than 7 inputs and 7 outputs. An analysis of positive and negative network connections revealed a bias towards positive outputs. The pretest probability of a positive outcome prediction is 0.66 and the pretest probability of a negative prediction is therefore 0.34. This system bias was used when applying the binomial test to all simulation outcomes.

### Simulation of the aiPSC model

Studies have shown that iPSCs express many factors that are consistent with the signature of undifferentiated human ES cells. These factors include, OCT3/4, SOX2, NANOG, growth and differentiation factor 3 (GDF3), reduced expression 1 (REX1), fibroblast growth factor 4 (FGF4), embryonic cell-specific gene 1 (ESG1/DPPA5), developmental pluripotency-associated 2 (DPPA2), DPPA4, and telomerase reverse transcriptase (hTERT) [6, 29]. It is also noteworthy that expression levels of OCT3/4, SOX2, NANOG, SALL4, E-CADHERIN and hTERT determined by western blotting and were similar in iPSC and hESC [6].

In this study we have programmed DeepNEU to simulate iPSCs (aiPSC) using defined sets of reprogramming factors. We have turned on the key transcription factors that were previously reported to induce pluripotency. Briefly, OCT3/4, SOX2, KLF4 and CMYC were turned on [5].

**Fig. 1:**
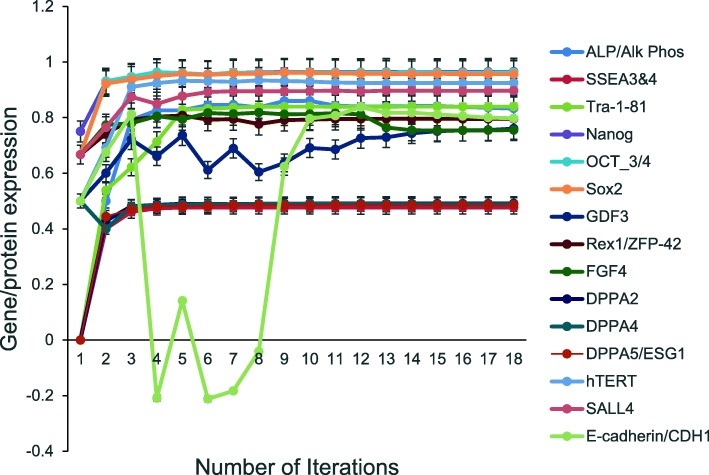
Expression of pluripotency factors by the aiPSC model. Unsupervised DeepNEU simulation of aiPSC model, which was experimentally validated by [5]. The model converged after 18 iterations and expressed the same human ESC surface antigens and undifferentiated ECS markers were also upregulated (*N* = 15, *p* = 0.002). Data are representative of three independent simulation experiments; e*rror bars* indicate ± SEM

The unsupervised aiPSC model converged quickly (18 iterations) to a new system wide steady state without evidence of overtraining after 1000 iterations. The aiPSC model expressed the same human ESC specific surface antigens, including SSEA-3/4, tumor-related antigen TRA-1-81, alkaline phosphatase (ALP) and NANOG protein. The current aiPSC system did not implement the tumor-related antigen TRA-1-60 and therefore it could not be evaluated. Interestingly, all the above mentioned undifferentiated ESC makers were also upregulated in the aiPSC model system. These ESC markers studied in iPSC were also elevated in the aiPSC model (Fig. 1). The probability that all (*N* = 15) pluripotency outcomes were predicted by chance alone using the binomial test is 0.002.

While the aiPSC model was not specifically designed to evaluate embryoid markers-mediated differentiation, it was possible to critically evaluate the same markers examined in [6] that were used to confirm line specific differentiation identified by immunocytochemistry and/or RT-PCR by [6] and summarized in Table 1 below.

**Table 1:** Embryoid markers-mediated differentiation expressed by aiPSCs

Ectodermal markers (*N* = 4)	Mesodermal markers (N = 6)	Endodermal markers (N = 5)
bIII-tubulin (TUBB3)	alpha-smooth muscle actin (a-SMA)	forkhead box A2 (FOXA2)
Glial fibrillary acidic protein (GFAP)	desmin, alpha-fetoprotein (AFP)	AFP
Microtubule-associated protein 2 (MAP2)	vimentin mesoderm	cytokeratin 8/18 (CK8/CK18)
Apaired box 6 (PAX6)	vimentin parietal endoderm	SRY-box containing gene 17 (SOX17)
	BRACHYURY	
	Msh homeobox 1 (MSX1)	

**Fig. 2:**
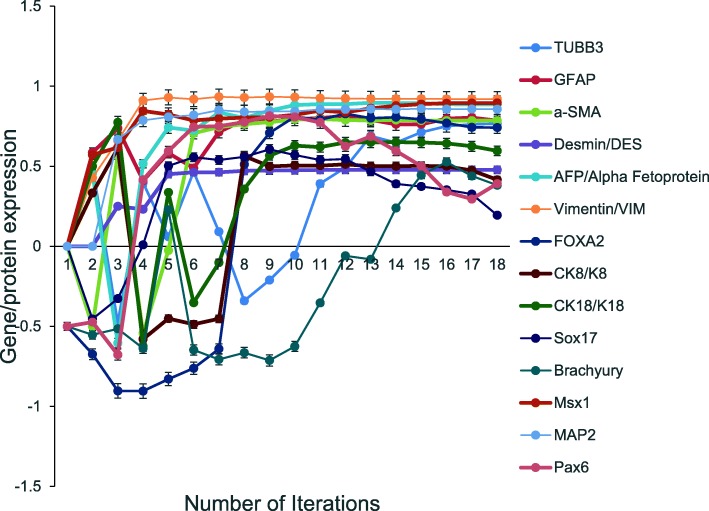
Differentiation potential of the aiPSC model. Embryonic markers-mediated differentiation were predicted to be expressed by aiPSC model as shown experimentally by [5]. Total of (*N* = 14) embryonic differentiation markers were expressed by aiPSC. Specifically, (*N* = 4) ectodermal markers, (*N* = 6) Mesodermal markers and (*N* = 5) endodermal markers (*p* = 0.003). Data are representative of three independent simulation experiments; e*rror bars* indicate ± SEM

All these genes were expressed/up regulated in the aiPSC system (Fig. 2). The probability that all (*N* = 14) of the line specific differentiation outcomes were predicted by chance alone using the binomial test is 0.003.

### The aiNSC model

**Fig. 3:**
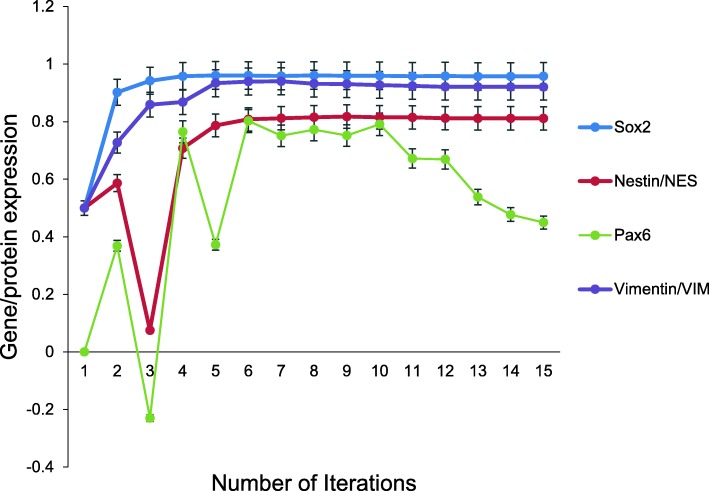
Expression of NSC markers by aiNSC. Unsupervised DeepNEU simulation of aiNSC model, which was experimentally validated by [27]. The model converged after 15 iterations and expressed NSC specific markers PAX6, NESTIN, VIMENTIN and SOX2. (N = 15, p = 0.002). Data are representative of three independent simulation experiments; e*rror bars* indicate ± SEM

**Fig. 4:**
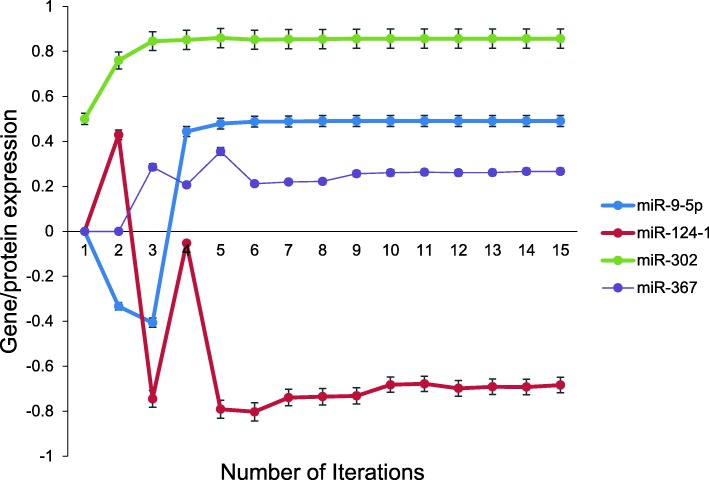
Expression of several miRNAs by aiNSC. aiNSC model also expressed several microRNAs, which were also evaluated by Yu et al, (2015). The expression levels of miR-9-5p, miR-302 and miR-367 were upregulated, but miR-124-1 was downregulated in aiNSC. (N = 15, p = 0.002). Data are representative of three independent simulation experiments; e*rror bars* indicate ± SEM

We next employed DeepNEU to generate the unsupervised aiNSC model by turning off LET7 and turning on SOX2 to convert human fibroblasts directly into induced neural stem cells (iNSC) Yu et al. [27]. The unsupervised aiNSC model converged quickly (15 iterations) to a new system wide steady state without evidence of overtraining after 1000 iterations. Like the hiNSC cellular model, the aiNSC simulation expressed several NSC specific markers including PAX6, NESTIN, VIMENTIN and SOX2 (Fig. 3). In addition, several microRNAs were also evaluated by Yu et al, (2015). The authors determined that the expression levels of miR-9-5p, miR-9-3p, and miR-124 were upregulated in the hiNSCs, but other miRNAs namely miR-302/miR-367 were not detected in their system. In the aiNSC simulation, miR-9-5p was also upregulated while miR-124 was down regulated. Unlike the hiNSC, the aiNSC expressed miR-302/miR-367 which were also “abundantly” expressed in hESC (Fig. 4). miR-9-3p was not implemented in the current version of the aiNSC simulation and therefore could not be evaluated.

**Fig. 5:**
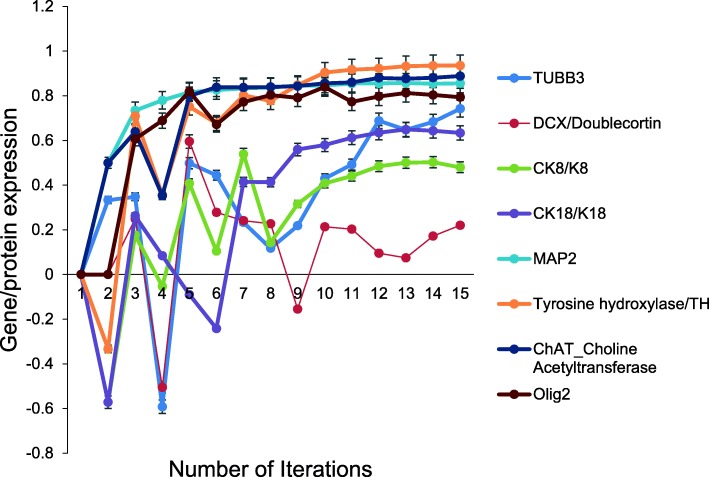
Expression of neuronal specific markers by aiNSC. Several early neuronal markers were expressed by the aiNSC simulation. Namely, CK18/K18, MAP2, TUBB3, DCX/Doublecortin, CK8/K8, TH, ChAT, and OLIG2 were all expressed in the aiNSC simulation. The probability that 16 of the 17 (94.12%) neuronal marker expression outcomes were accurately predicted by chance alone using the binomial test is (*p* = 0.0075). Data are representative of three independent simulation experiments; e*rror bars* indicate ± SEM

Next, Yu et al. [27] demonstrated that the hiNSC could be differentiated into neurons, astrocytes and oligodendrocytes, the three main neural lineages. Immunohistochemistry was used to demonstrate the expression of specific early neuronal markers including class III beta-tubulin (TUJ1/TUBB3), doublecortin (DCX) and neuronal intermediate filaments. Cytokeratin 8 and 18 (CK8/CK18) were the neuronal intermediate fibers implemented in the aiNSC while a-internexin was not implemented in this version of the aiNSC. Several early neuronal markers were also expressed by the aiNSC simulation. Subsequently, the mature neuronal marker, MAP2; the dopaminergic and noradrenergic neuron marker, tyrosine hydroxylase (TH); the cholinergic neuron marker, choline acetyltransferase (ChAT); the astrocyte marker, Glial fibrillary acidic protein (GFAP); and the oligodendrocyte marker, OLIG2 were all expressed in the aiNSC simulation (Fig. 5). The O4 oligodendrocyte marker was not implemented in this version of the aiNSC. The probability that 16 of the 17 (94.12%) neuronal marker expression outcomes were accurately predicted by chance alone using the binomial test is 0.0075.

**Fig. 6:**
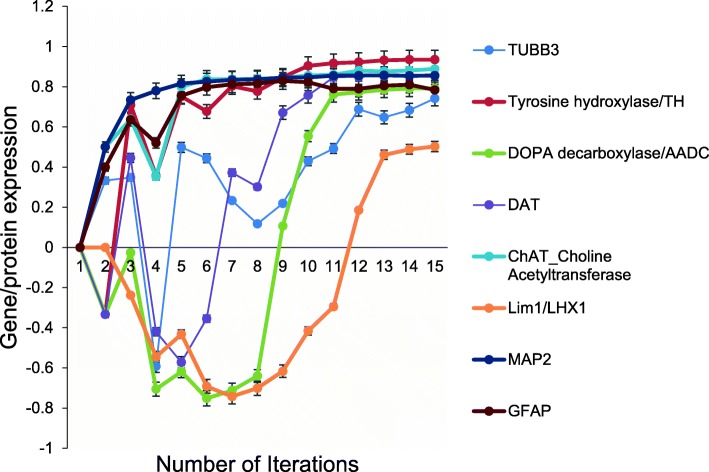
Neuronal Markers identified by Takahashi et al., (2007) and expressed by aiNSC. All markers identified in hNSC by Takahashi et al (2007) in addition to GFAP were also predicted to express in aiNSC model. (*N* = 8, *p* = 0.036). Data are representative of three independent simulation experiments; e*rror bars* indicate ± SEM

Takahashi et al. [5, 6] also directed differentiation of hiPSC into neural cells. Immunocytochemistry was used to confirm expression of TH and TUBB3 by differentiating cells. PCR analysis revealed expression of dopaminergic neuron markers, dopa-decarboxylase (AADC) and member 3 (DAT); ChAT; LIM homeobox transcription factor 1 beta (LMX1B); and the mature neuron marker, MAP2. However, the astrocyte marker, GFAP was not expressed in their system. All markers identified by Takahashi et al. [5, 6] plus GFAP were expressed in the aiNSC simulation (Fig. 6). The probability that these neuronal marker expression outcomes (*N* = 8) were predicted by chance alone using the binomial test is 0.036.

### The aiCMC (cardiomyocyte) model

**Fig. 7:**
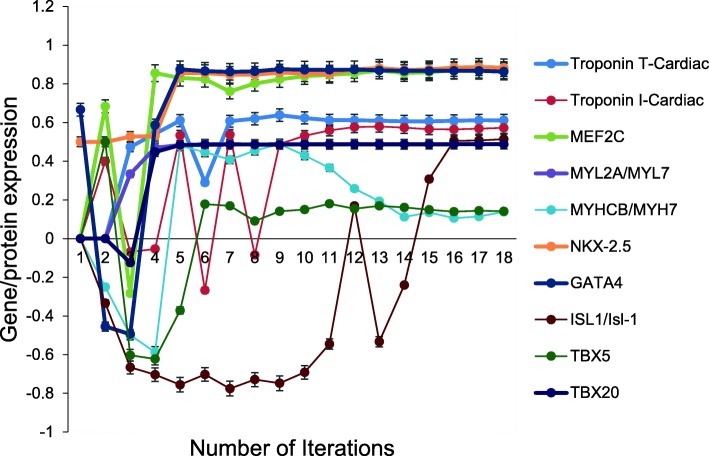
Expression of cardiomyocyte markers by aiCMC. Unsupervised DeepNEU simulation of aiCMC model, which was experimentally validated by [30]. The model converged after 15 iterations and expressed iCMC specific markers consistent with [30]. (*N* = 10, *p* = 0.016). Data are representative of three independent simulation experiments; e*rror bars* indicate ± SEM

A protocol adding Activin A and member of the bone morphogenetic protein 4 (BMP4) to the generation of generic aiPSC resulted in an aiCMC model that converged after 15 iterations without evidence of overtraining after 1000 iterations. Takahashi et al. [5, 6] used a similar protocol to successfully direct the differentiation of hiPSC into clumps of beating cells. RT-PCR showed that these cells expressed cardiomyocyte markers including troponin T type 2 cardiac (TnTc); myocyte enhancer factor 2C (MEF2C); regulatory myosin light polypeptide 2A (MYL2A); myosin, heavy polypeptide 7 cardiac muscle beta (MYHCB); and NK2 transcription factor-related locus 5 (NKX2.5) [6]. All the cardiomyocyte markers above were also expressed by the aiCMC system (Fig. 7). Five additional cardiomyocyte markers identified in [30] including, GATA-4, Isl-1, Tbx-5, Tbx-20 and cardiac Troponin I were also expressed by the aiCMC system. The probability that the cardiomyocyte marker expression outcomes (*N* = 10) were predicted by chance alone using the binomial test is 0.016.

### An aiNSC for simulating Rett syndrome (MeCP2 deficiency)

**Fig. 8:**
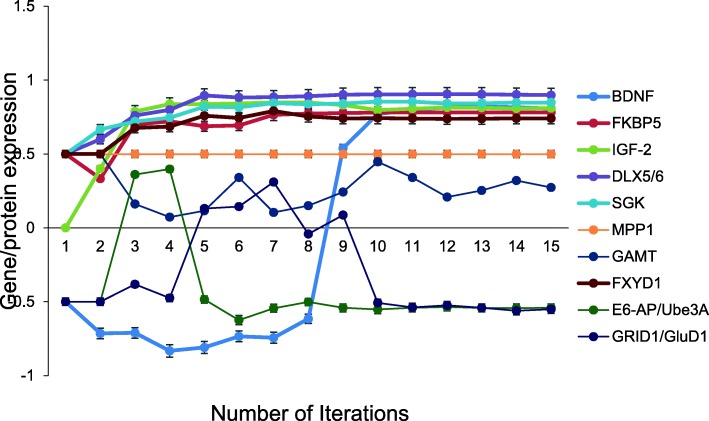
Expression profile of RETT neuron by aiNSC. Unsupervised aiNSC model was programmed with the gene MeCP2 locked off to simulated RETT syndrome. The model converged after 18 iterations to generate RETT neuron as reported in [26]. Specifically, BDNF, FKBP5, IGF2, DLX5, DLX6, SGK1, MPP1, GAMT and FXYD were upregulated, while genes UBE3A and GRID1/GluD1 were both downregulated. (*N* = 11, p = 0.01). Data are representative of three independent simulation experiments; e*rror bars* indicate ± SEM

Finally, we have used our unsupervised aiNSC model that was validated based on the Yu et al. [27] recipe for direct conversion of human fibroblasts to iNSC with the gene MeCP2 locked off to simulate a Rett syndrome neuron. The model converged quickly (15 iterations) to a new system wide steady state without evidence of overtraining after 1000 iterations. The actual Rett neuron(s) generated and evaluated in [26] had the following gene expression profile. The upregulated genes were Brain-derived neurotrophic factor (BDNF), FKBP5, Insulin-like growth factor II (IGF2), Distal-Less Homeobox 5 (DLX5), Distal-Less Homeobox 6 (DLX6), Serine/threonine-protein kinases 1 (SGK1), Membrane Palmitoylated Protein 1 (MPP1), Guanidinoacetate N-Methyltransferase (GAMT) and Gene coding Phospholemman (FXYD1) while genes Ubiquitin-protein ligase E3A (UBE3A) and Glutamate Ionotropic Receptor Delta Type Subunit 1 (GRID1/GluD1) were both downregulated. All up and down gene regulation predictions from the aiNSC-Rett neuron simulation were correct and these data are presented in [26](Fig. 8). The probability that all (*N* = 11) of the Rett neuron specific outcomes were predicted by chance alone using the binomial test is 0.01.

## Discussion

The use of hSCs in medicine is limited by the abundance of/accessibility to somatic cells from a donor and histocompatibility Issues with donor/recipient transplants. These two factors largely determine the reliability of hSCs for drug development and developmental studies. Nevertheless, the development of iPSCs from donor somatic cells has proven to be somewhat successful. Issues of histocompatibility with donor/recipient transplants that have been reported with hESCs and adult stem cells (ASCs) can be avoided. Additionally, information gathered from the reprogramming process that results in iPSCs is very promising for drug development research of rare diseases and developmental studies [31]. Unfortunately, the application of iPSCs is also hindered by the highly variable efficiency of SC induction protocols and the significant costs that leads to uncertainty because of reduced reproducibility and long-term maintenance of iPSCs. In this study, we introduced an efficient, accurate, cost-effective and highly customizable computational platform to enable aiPSC model generation.

An increasing number of studies have employed computational, statistical, and mathematical approaches for modelling and analyzing the underling factors that regulate cellular reprogramming. These efforts have largely focused on specific elements of cellular reprogramming. Examples of this previous work include, (1) a Bayesian network model (i.e., a probabilistic model) provided conditional analysis of random signaling network interactions [32], (2) a Boolean network model (i.e.*,* a quantitative model) was used to study the logical interactions of network components [33], (3) a multi-scale model, in which a framework of combined algorithms was used to mathematically predict effects of factors/genes on other factors/genes [34], (4) a clustering algorithm, in which multiple algorithms were used to organize data points into groups that share certain similarities to enable mathematical modeling and simulation of cellular events [35] and (5) a Support Vector Machine learning model (SVM), in which a fully supervised computational approach was used to classify datasets into pre-defined categories to enable phenotypic profiling of cellular subsets [36, 37]. A more in-depth review of computational tools used in stem cell research has been published recently [38].

Unlike previous and largely supervised models focused on various aspects of cellular reprogramming, the unsupervised DeepNEU platform provides a novel high dimensional and nonlinear approach for simulating simple aiPSCs, and to qualitatively assess stem cell regulatory mechanisms and pathways using a literature validated set of reprogramming factors in the context of a fully connected hybrid RNN. Once validated with the results of peer reviewed wet-lab experiments, DeepNEU aiPSC models provide an efficient, programmable, and cost-effective tool for empowering rare disease and other researchers.

In this research work, the performance of the DeepNEU platform (Version 3.2) was evaluated extensively through simulation of several experimentally validated iPSC models including iPSCs, iNSCs, iCMCs and a Rett syndrome model using aiNSC with MeCP2 deficiency.

DeepNEU simulation of aiPSCs showed that the gene expression profiles of the simulated cells were consistent with that of iPSCs. aiPSCs express many factors that are consistent with the signature of undifferentiated human ES cells. These factors include, OCT3/4, SOX2, NANOG, growth and differentiation factor 3 (GDF3), reduced expression 1 (REX1), fibroblast growth factor 4 (FGF4), embryonic cell-specific gene 1 (ESG1/DPPA5), developmental pluripotency-associated 2 (DPPA2), DPPA4, and telomerase reverse transcriptase (hTERT) [6, 29]. Additionally, the unsupervised DeepNEU successfully simulated embryoid body-mediated differentiation (see Table 1) to confirm line specific differentiation identified by immunocytochemistry and/or RT-PCR in Takahashi et al. [5, 6].

The unsupervised aiNSCs model (Fig. 3) showed that the gene/protein expression profile was consistent with the hiNSC cellular model. The aiNSC simulation also expressed several NSC specific markers including PAX6, NESTIN, VIMENTIN and SOX2.

In the study conducted by Yu et al. [27] the expression levels of miR-9-5p, miR-9-3p, and miR-124 were upregulated in the hiNSCs but other miRNAs, namely miR-302/miR-367, were not detected in their system. Interestingly in our simulated aiNSC model miR-9-5p was also upregulated while miR-124 was downregulated. Unlike the hiNSC, the aiNSC expressed miR-302/miR-367 which were also “abundantly” expressed in human embryonic stem cells (hESC) (Fig. 4).

On the other hand, PCR analysis revealed expression of dopaminergic neuron markers, dopa-decarboxylase (AADC) and member 3 (DAT); ChAT; LIM homeobox transcription factor 1 beta (LMX1B); and the mature neuron marker, MAP2 (Takahashi et al, 2007). However, the astrocyte marker, GFAP was not expressed in their system. All markers identified by Takahashi et al. [5, 6] plus GFAP were expressed in the aiNSC simulation (Fig. 6).

All the cardiomyocyte markers that were reported to be expressed by iCMCs were also expressed in the unsupervised aiCMC system (Fig. 7) entirely consistent with the data provided by Takahashi et al. [5, 6]. Five additional cardiomyocyte markers identified in Rajala et al. (2012) including GATA-4, Isl-1, Tbx-5, Tbx-20 and cardiac Troponin I were also expressed by the aiCMC system.

### DeepNEU to simulate rare disease− aiNSC for simulating RETT syndrome (MeCP2 deficiency)

To validate DeepNEU platform efficiency in modeling a rare disease (RETT syndrome) was simulated using the aiNSC protocol with the MeCP2 gene locked off. Interestingly, the upregulated genes were BDNF, FKBP5, IGF2, DLX5, DLX6, SGK1, MPP1, GAMT and FXYD1 while genes UBE3A and GRID1/GluD1 were both downregulated. All up and down regulated genes in the aiNSC-RETT neuron simulation are entirely consistent with the expression data presented in Ehrhart et al. [26] (Fig. 8).

To the best of our knowledge, this is the first-time computer simulations of intact and functioning iPSC have been successfully used to accurately reproduce the landmark experimental results reported by Takahashi et al. (2007) and other studies cited above. The technology itself has limited overlap with some features of neutrosophic cognitive maps, evolutionary systems, neural networks and SVM applied to create a novel unsupervised machine learning platform. The papers referenced above were the source for the reprogramming and media factors used to construct the input vector for the simulations. These papers were also used here to validate in an unsupervised manner the genotypic and phenotypic output features of the simulation at the new stable state.

## Conclusion/Significance

Stem cell research will inevitably be transformed by computer technologies. The results of the initial DeepNEU project indicate that currently available stem cell data, computer software and hardware are sufficient to generate basic artificially induced pluripotent stem cells (aiPSC). These initial DeepNEU stem cell simulations accurately reproduced gene and protein expression results from several peer reviewed publications.

The application of this computer technology to generate disease specific aiPSCs has the potential to improve (1) disease modeling, (2) rapid prototyping of wet lab experiments, (3) grant application writing and (4) specific biomarker identification in a highly cost-effective manner. Further development and validation of this promising new technology is ongoing with the current focus on modelling rare genetic diseases.

## Methods

DeepNEU platform: We have developed a novel and powerful deep-machine learning platform employing a fully-connected recurrent neural network (RNN) architecture, in which each of the inputs is connected to its output nodes (feedforward neurons) and each of the output nodes is also connected back to their input nodes (feedback neurons). There are at least two major benefits of using this network architecture. First, RNN can use the feedback neurons connections to store information over time and develop “memory”. Second, RNN networks can handle sequential data of arbitrary length [39]. For example, RNN can be programmed to simulate the relationship of a specific gene/protein to another gene/protein (one to one), gene/protein to multiple genes/proteins (one to many), multiple genes/proteins to one gene/protein (many to one) and multiple genes/proteins to different multiple genes/proteins (many to many). Our novel RNN DeepNEU network was developed with one network processing layer for each input to promote complex learning and analysis of how different genes and pathways are potentially regulated in embryonic and reprogrammed somatic cells in key signaling pathways. Here we have used DeepNEU to simulate aiPSCs by using defined sets of reprogramming factors (genes/proteins were turned on or off based on the modeled iPSCs)*.*

### Dataset

We have incorporated into the DeepNEU database key genes/proteins that were reported to be involved in regulating and maintaining signaling pathways in human embryonic stem cells (hESCs) and induced human pluripotent stem cells (hiPSCs). We have gathered genes/proteins based on literature reports that extensively studied cellular pathways of hESC and/or hiPSC [40–49]. Abundant data were available. For example, a PubMed (PMC) search of the literature with “stem cells” returned more than 435,000 hits. A more focused query using “stem cell signaling”, returned more than 261,000 hits. Nevertheless, data that were included in the DeePNEU database were selected with a preference for (1) human stem cell data, (2) recency of peer reviewed English language publications and (3) highest impact factors of the journals under consideration.

To that end, the data was used to create a list of important genes/proteins (data not shown) based on their documented contributions to human stem cell signaling pathways. The current version of the database includes 3589 gene/protein (inputs) involved in hESC cellular pathways and 27,566 gene/protein regulatory relationships important in hESC that were used for aiPSC system modelling. Importantly, this simple data representation permits complex relationships including both positive and negative feedback loops that are common in biological systems.

### Entry of data to DeepNEU database

All data (genes/proteins, and relationships) were entered, formatted and stored as a large CSV (comma separated values) file in Delimit Professional (v3.7.5, Delimitware, 2017). This database manager was chosen because it can efficiently handle very large CSV files where data can be represented as an NxN (an array of values with N rows and N columns) relationship matrix. In addition, built-in data entry and file scan functions help to ensure and maintain data integrity. This software can also import and export multiple data file types facilitating two-way interaction with a wide range of data analysis tools. Finally, the software scales easily to NxN or NxM (an array of values with N rows and M columns) databases having millions of rows and columns (http://delimitware.com, 2017).

### DeepNEU platform

The DeepNEU platform uses a novel, but powerful neutrosophic logical (NL) framework to represent relationships between signaling genes/proteins. NL was originally created by Florentin Smarandache in 1995. In NL, every logical variable X is described by an ordered triple, X = (T, I, F) where T is the degree of truth, “I” is the degree of indeterminacy, and F is the degree of false. The strength of any relationship can have any real value between − 1 and + 1 or “I” if the relationship is considered indeterminate. Positive or stimulatory causal relationships are represented by + 1 in the database unless there is a fractional value > 0 and < = + 1. Similarly, negative or inhibitory causal relationships are represented by − 1 in the database unless a fractional value < 0 and > = − 1 is provided. Relationships are considered indeterminate and represented by an “I” if multiple sources report conflicting data or if the relationship is labelled with a question mark in an associated process flow diagram. A value of zero is used when no relationship between nodes is known or suspected [50]. NL is an extension and generalization of Fuzzy Logic and can be easily converted by replacing all indeterminate (I) relationships with zeros (i.e. by assuming there is no causal relationship).

### DeepNEU network architecture

The NxN relationship matrix is the core data for an unsupervised fully-connected RNN. A learning system is referred to as supervised when each data pattern is associated with a specific numerical (i.e., regression) or category (i.e., classification) outcome. Unsupervised learning is used to draw inferences from datasets consisting of input data patterns that do not have labeled outcomes [50]. DeepNEU is a complex learning system in that every (gene/protein) node in the multilayered network is connected to every other node in the network. Traditional neural networks have one or a few hidden or processing layers between the input layer and the output layer. Advanced deep-learning neural networks can have more than a dozen processing layers [51, 52]. DeepNEU has one processing layer for each input variable. Taken together, the input variables and their declared initial values constitute an N-dimensional initial input vector. Vector-Matrix multiplication uses this N-dimensional input vector and the NxN relationship matrix to produce an N-dimensional output or new state vector. The new state vector becomes the new input vector for the next iteration and this iterative process continues until a new system wide steady state is achieved. In general terms, the DeepNEU network architecture is similar to Neutrosophic and Fuzzy Cognitive Maps (NCMs/FCMs; used to represent causal relationship between concepts (genes/proteins)) which are also examples of fully-connected and recurrent neural networks [53, 54].

### The DeepNEU simulations

The initial goal of this project was to first create a computer simulation of a hiPSC and then validate the model using the results published by Takahashi et al. in 2007 and others as described above. Briefly, the input or initial state vector of dimension N was set to all zeros except for transcription factors OCT3/4, SOX2, KLF4 and CMYC. These four factors were given a value of + 1 indicating that they were turned on for the first iteration. These values were not locked on so that all subsequent values were determined by system behavior.

### DeepNEU simulation protocol

1. The machine learning process began with vector matrix multiplication (VMM). The NxN relationship matrix was multiplied by the “N”-dimensioned input vector with OCT3/4, SOX2, KLF4 and CMYC turned on. Both the input vector and relationship matrix are comprised mostly of zeros. The input vector and relationship matrix were both considered to be sparse. To minimize the computational burden, sparse vector matrix multiplication algorithms were employed at each iteration during model generation.
2. At each iteration the sparse VMM operation produces an “N”-dimensional output vector with variable components many of which have large positive or negative values. To avoid computational explosion a squashing or activation function was used to map these values between a minimum of − 1 and a maximum of + 1. After initial evaluation of several activation functions, the Elliott function was selected based on rapidity of system convergence and outcome reproducibility [55]. At the end of the activation process, the squashed N-dimensional output vector becomes the new input vector for the next iteration. This cycle is repeated until system convergence occurs indicating that a new system wide steady state has been achieved.
3. The goal of the learning system is to minimize error. In this case the error being considered is the mean squared error (MSE) between a given output vector and the previous output vector. During model development several error functions including adjusted R^2^, SVM/Vapnik loss and MSE were evaluated. The MSE function was selected because its’ use consistently resulted in faster system convergence and more reproducible results. While the MSE function has been widely used it has also been widely criticized because the function can perform poorly due to squaring in the presence of outliers. In the current project, the error function was applied after the raw system output was “squashed” between values of − 1 and + 1 using a sigmoid type function. This squashing effectively mitigates the problem of potential outliers. As learning continues the MSE converges towards zero. For this project system convergence was defined at MSE < 0.001 and model generation stops. The system output is then saved as a CSV data file for further analysis.
4. The final output from the aiPSC model regarding the expression or repression of genes and proteins was directly compared with published expression profiles [6]. Model prediction values > 0 were classified as expressed or upregulated while values < 0 were classified as not expressed or downregulated. Statistical analysis of the aiPSC predictions and the published data used the Binomial Test. This test provides an exact probability, can compensate for prediction bias and is ideal for determining the statistical significance of experimental deviations from an actual distribution of observations that fall into two outcome categories (e.g., agree vs disagree). A *p*-value < 0.05 is considered significant and is interpreted to indicate that the observed relationship between aiPSC predictions and actual outcomes is unlikely to have occurred by chance alone.

## Abbreviations

AADC: Dopa-decarboxylase
AFP: Desmin, alpha-fetoprotein
aiCMCs: Artificially-induced cardiomyocytes
aiNSCs: Artificially-induced neural stem cells
aiPSC: Artificially-induced pluripotent stem cells
ALP: Alkaline phosphatase
ASCs: Adult stem cells
a-SMA: Alpha-smooth muscle actin
BDNF: Brain-derived neurotrophic factor
BMP4: Member of the bone morphogenetic protein 4
ChAT: Choline acetyltransferase
CK8/CK18: Cytokeratin 8/18
CSV: Comma separated values
DAT: Member 3
DCX: Doublecortin
DLX5: Distal-Less Homeobox 5
DLX6: Distal-Less Homeobox 6
DPPA2: Developmental pluripotency-associated 2
DPPA4: Developmental pluripotency-associated 4
DPPA5: Developmental pluripotency-associated 5
ESG1: Embryonic cell-specific gene 1
FCM: Fuzzy cognitive map
FGF4: Fibroblast growth factor 4
FOXA2: Forkhead box A2
FXYD1: Gene coding phospholemman
GAMT: Guanidinoacetate N-Methyltransferase
GATA-4: Critical transcription factor for proper mammalian cardiac development
GDF3: Growth and differentiation factor 3
GFAP: Glial fibrillary acidic protein
GRID1/GluD1: glutamate Ionotropic Receptor Delta Type Subunit 1.
hESCs: Human embryonic stem cells
hPSC: Human pluripotent stem cells
hTERT: Telomerase reverse transcriptase
IGF2: Insulin-like growth factor II
iPSCs: Induced pluripotent stem cells
LMX1B: LIM homeobox transcription factor 1 beta
MAP2: Microtubule-associated protein 2
MeCP2: Methyl-CpG-binding protein 2
MEF2C: Myocyte enhancer factor 2C
miR: microRNAs
MPP1: Membrane palmitoylated Protein 1
MSE: Mean squared error
MSX1: Msh homeobox 1
MYHCB: Myosin, heavy polypeptide 7 cardiac muscle beta
MYL2A: Myosin, light polypeptide 2A,
NCM: Neutrosophic cognitive map
NKX2.5: NK2 transcription factor-related locus 5
NL: Neutrosophic logical
OLIG2: Oligodendrocyte transcription factor 2
PAX6: Apaired box 6
REX1: Reduced expression 1
RNN: Recurrent neural network
SCNT: Somatic cell nuclear transplantation
SGK1: Serine/threonine-protein kinases 1
SOX17: SRY-box containing gene 17
SSEA-3/4: Specific surface antigens3/4
TH: Tyrosine hydroxylase
TnTc: Troponin T type 2 cardiac
TRA-1-60: Tumor-related antigen-1-60
TRA-1-81: Tumor-related antigen-1-81
TUBB3: bIII-tubulin
UBE3A: Ubiquitin-protein ligase E3A
VMM: Vector matrix multiplication
